# A Narrative Review on LI-RADS Algorithm in Liver Tumors: Prospects and Pitfalls

**DOI:** 10.3390/diagnostics12071655

**Published:** 2022-07-07

**Authors:** Federica De Muzio, Francesca Grassi, Federica Dell’Aversana, Roberta Fusco, Ginevra Danti, Federica Flammia, Giuditta Chiti, Tommaso Valeri, Andrea Agostini, Pierpaolo Palumbo, Federico Bruno, Carmen Cutolo, Roberta Grassi, Igino Simonetti, Andrea Giovagnoni, Vittorio Miele, Antonio Barile, Vincenza Granata

**Affiliations:** 1 Department of Medicine and Health Sciences V. Tiberio, University of Molise, 86100 Campobasso, Italy; demuziofederica@gmail.com; 2 Division of Radiology, Università degli Studi della Campania Luigi Vanvitelli, 81100 Naples, Italy; francesca.grassi1@studenti.unicampania.it (F.G.); federica.dellaversana@studenti.unicampania.it (F.D.); roberta.grassi@policliniconapoli.it (R.G.); 3 Medical Oncology Division, Igea SpA, 80013 Naples, Italy; 4 Division of Radiology, Azienda Ospedaliera Universitaria Careggi, 50134 Florence, Italy; ginevra.danti@gmail.com (G.D.); federicaflammia91@gmail.com (F.F.); giudittachiti@gmail.com (G.C.); vmiele@sirm.org (V.M.); 5 Italian Society of Medical and Interventional Radiology (SIRM), SIRM Foundation, 20122 Milan, Italy; palumbopierpaolo89@gmail.com (P.P.); federico.bruno.1988@gmail.com (F.B.); 6 Department of Clinical Special and Dental Sciences, University Politecnica delle Marche, 60126 Ancona, Italy; t.valeri@univpm.it (T.V.); a.agostini@staff.univpm.it (A.A.); a.giovagnoni@univpm.it (A.G.); 7 Department of Radiological Sciences, University Hospital Ospedali Riuniti, Via Tronto 10/a, 60126 Torrette, Italy; 8 Area of Cardiovascular and Interventional Imaging, Department of Diagnostic Imaging, Abruzzo Health Unit 1, 67100 L’Aquila, Italy; 9 Emergency Radiology, San Salvatore Hospital, Via Lorenzo Natali 1, 67100 L’Aquila, Italy; abarile63@gmail.com; 10 Department of Medicine, Surgery and Dentistry, University of Salerno, 84084 Fisciano, Italy; carmencutolo@hotmail.it; 11 Radiology Division, Istituto Nazionale Tumori-IRCCS-Fondazione G. Pascale, Via Mariano Semmola, 80131 Naples, Italy; igino.simonetti@istitutotumori.na.it (I.S.); v.granata@istitutotumori.na.it (V.G.)

**Keywords:** liver, diagnosis, LI-RADS

## Abstract

Liver cancer is the sixth most detected tumor and the third leading cause of tumor death worldwide. Hepatocellular carcinoma (HCC) is the most common primary liver malignancy with specific risk factors and a targeted population. Imaging plays a major role in the management of HCC from screening to post-therapy follow-up. In order to optimize the diagnostic-therapeutic management and using a universal report, which allows more effective communication among the multidisciplinary team, several classification systems have been proposed over time, and LI-RADS is the most utilized. Currently, LI-RADS comprises four algorithms addressing screening and surveillance, diagnosis on computed tomography (CT)/magnetic resonance imaging (MRI), diagnosis on contrast-enhanced ultrasound (CEUS) and treatment response on CT/MRI. The algorithm allows guiding the radiologist through a stepwise process of assigning a category to a liver *observation*, recognizing both major and ancillary features. This process allows for characterizing liver lesions and assessing treatment. In this review, we highlighted both major and ancillary features that could define HCC. The distinctive dynamic vascular pattern of arterial hyperenhancement followed by washout in the portal-venous phase is the key hallmark of HCC, with a specificity value close to 100%. However, the sensitivity value of these combined criteria is inadequate. Recent evidence has proven that liver-specific contrast could be an important tool not only in increasing sensitivity but also in diagnosis as a major criterion. Although LI-RADS emerges as an essential instrument to support the management of liver tumors, still many improvements are needed to overcome the current limitations. In particular, features that may clearly distinguish HCC from cholangiocarcinoma (CCA) and combined HCC-CCA lesions and the assessment after locoregional radiation-based therapy are still fields of research.

## 1. Introduction

Liver cancer is the sixth most detected tumor and the third leading cause of tumor death worldwide [1,2,3,4,5,6,7]. Hepatocellular carcinoma (HCC) is the most common primary liver malignancy (more than 80%), with a higher prevalence in male patients (3:1 M/F) and in developing countries (72% in Asia compared to 9.8% and 5% in Europe and the USA, respectively) [2]. At present, chronic liver disease is the main cause of HCC development, with viral cirrhosis remaining the primary cause of carcinogenesis [8]. Indeed, HBV and HCV chronic infection are still responsible for 56 and 20% of deaths from liver cancer, respectively [1,2]. However, in the last few decades, due to an increase in obesity and type 2 diabetes, also nonalcoholic fatty liver disease (NAFLD) is becoming an emerging cause of HCC (annual incidence of approximately 2.4–12.8%), especially in the Western world [9,10,11,12,13,14]. Other risk factors include environmental exposure to aflatoxins, anabolic steroids, oral contraceptives and tobacco and alcohol abuse [1]. Finally, albeit in a small percentage of cases, some benign lesions such as adenomas (5% of all cases) may undergo neoplastic degeneration [15,16,17].

Imaging plays a major role in the management of HCC from screening to post-therapy follow-up [18,19,20,21,22,23,24,25,26,27,28,29,30,31,32,33,34,35,36,37,38,39]. In particular, ultrasonography (US) is the method of choice for tumor screening, while the role of computed tomography (CT) and magnetic resonance imaging (MRI) for HCC diagnosis and post-treatment assessment is largely consolidated in clinical practice [3,40,41,42,43,44,45,46,47,48,49,50,51,52,53,54,55,56,57,58,59,60,61].

In order to optimize diagnostic accuracy and using a universal report that allows more effective communication among the multidisciplinary team caring for the patient, several classification systems have been proposed over time. Of these, the most valid and comprehensive is the Liver Imaging Reporting and Data System (LI-RADS), which provides guidance on all aspects of HCC imaging, from techniques for imaging acquisition to assessing treatment response and directing management. The latest version of ACR LI-RADS includes four algorithms: *(a)* US-LI-RADS for HCC screening and surveillance; *(b)* contrast-material-enhanced US for HCC diagnosis (CEUS-LI-RADS); *(c)* CT/MRI for HCC diagnosis and radiologic T stage (CT/MRI LI-RADS); and *(d)* CT/MRI for HCC treatment response assessment (TR LI-RADS) [62,63,64,65]. This system was conceived to emphasize positive predictive value and specificity in the diagnosis of HCC. On the other hand, unlike other diagnostic algorithms, LI-RADS includes an LR-M category that aims to preserve specificity and increase sensitivity in the definition of malignant lesions other than HCC [12,16]. Non-HCC malignancies include a broad spectrum of pathologies and some of them, such as intrahepatic cholangiocarcinoma (iCCA) or combined hepatocellular and cholangiocarcinoma (cHCC-CCA), are being recognized in patients with the same risk factors of HCC (chronic hepatitis or cirrhosis) [16,23,39]. Thereby, the correct differential diagnosis is essential for appropriately targeting treatment.

The aim of this narrative review was to evaluate the main current applications of LI-RADS in liver cancer, primarily in HCC and to assess the main radiological features that can guide differential diagnosis.

## 2. LI-RADS

LI-RADS is a system supported by the American College of Radiology (ACR), resulting from the work of radiologists experienced in liver pathology and integrated with the latest guidelines of the American Association for the Study of Liver Diseases (AASLD) [66]. The first version, proposed in 2011, aimed to standardize imaging and reporting on CT and MRI examinations in patients at risk of HCC. Based on scientific evidence, this version has been updated introducing other algorithms, which cover the entire diagnostic and therapeutic management of HCC patients [3].

Currently, LI-RADS comprises four algorithms addressing screening and surveillance, diagnosis on CT/MRI, diagnosis on contrast-enhanced ultrasound (CEUS) and treatment response on CT/MRI. Each algorithm has a core document that includes relevant indications to be applied in clinical practice [64]. A specific manual (LI-RADS manual) details the complex aspects of liver diseases, including imaging parameters, reporting instructions, templates and management recommendations. In order to adopt a universal language both in daily clinical practice and research, LI-RADS has published even a standardized lexicon, simplifying the communication between the different experts involved in HCC management [64,67]. The main focus of the LI-RADS system is to achieve high specificity and positive predictive value (PPV) in HCC detection and diagnosis, avoiding false-positive examinations. To ensure a high PPV, LI-RADS should be employed only in populations with a high pre-test probability of disease [3]. At present, LI-RADS and the American Association for the Study of Liver Diseases (AASLD) are in agreement in defining the target population at risk of HCC as those adult patients (≥18 years old) with cirrhosis, in the absence of current or prior HCC (incidence of HCC exceeds 1.5%/year) and subsets of patients with chronic HBV infection in whom the incidence of HCC exceeds 0.2% per year. In addition to the criteria for defining the high-risk screening population, patients with evidence of HCC or previous HCC are included in the diagnostic algorithm [67].

Both US-LI-RADS and diagnostic LI-RADS do not address patients with vascular causes of liver cirrhosis (e.g., cardiac hepatopathy, Budd–Chiari syndrome) because these conditions are associated with hypervascular benign liver lesions, which increase the risk of false positives and reduce the PPV for the diagnosis of HCC [67,68,69,70,71,72,73,74,75,76,77,78,79,80,81]. Although with some regional differences, patients with hepatitis C in the absence of cirrhosis and adults with nonalcoholic steatohepatitis are not included in the screening. Furthermore, patients in Child–Pugh class C who are not candidates for transplantation are excluded, considering their poor life expectancy [2].

An important point of the LI-RADS philosophy is the choice to adopt the term “*observation*” when referring to identified liver formations during the screening or diagnostic phase [67]. The generic term “*observation*” embraces the whole spectrum of possible abnormalities, ranging from simple cysts to clear neoplastic lesions [82,83,84,85,86,87,88,89,90,91,92,93,94]. In addiction, LI-RADS guidelines suggest the imaging techniques that could be used, according to the different diagnostic phases and the availability. At present, CT and MRI are the most widely used imaging modalities in the Western world, while the Asian-Pacific, Japanese and Chinese guidelines recommend CEUS for the diagnosis of HCC [95].

### 2.1. US-LI-RADS

The main purpose in the management of patients with HCC is to detect the disease at an early stage, when it is potentially treatable by resection or liver transplantation. Indeed, the average 5-year survival of advanced and untreated cases is only 15% [96]. US is proposed as a tool for cancer screening, and it has already been widely demonstrated in other oncological settings [97,98,99,100]. With regard to HCC, several studies showed the benefits in terms of reduced mortality and median survival of using US surveillance, particularly every six months in patients at risk of HCC with or without alpha-fetoprotein assay [101]. US shows a sensitivity in detecting HCC at an early stage of 63% and at any stage of 78–94% [102] and a specificity of 89% [103].

The ACR working group developed a standardized algorithm for US screening and surveillance in high-risk patients (US-LI-RADS) [67,104]. US-LI-RADS provides the classification of liver *observations* into three categories according to the probability of HCC, including an evaluation of the overall quality of the examination to define its reliability [67,104].

#### 2.1.1. Technique

**Figure 1:**
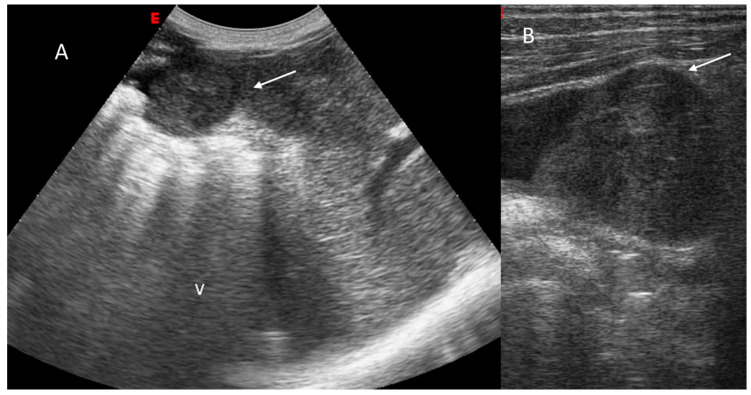
HCC on VIII seg. B mode assessment (**A**,**B**). The lesion shows inhomogeneous structure (arrow).

**Figure 2:**
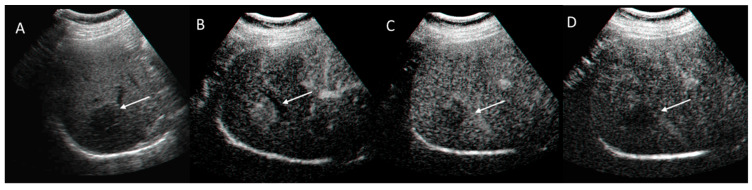
HCC on VII seg. US (**A**) and CEUS assessment. The lesion (arrow) shows APHE during arterial phase (**B**) with washout during portal (**C**) and late (**D**) phase of contrast study.

In order to achieve high diagnostic accuracy, the ACR US-LI-RADS working group illustrates some guidance on the technical execution of the US examination [104,105,106,107,108]. The examination should be performed using primarily a convex probe (Figure 1 and Figure 2), possibly combined with a linear transducer to explore the liver margins. Technical parameters have to be adjusted considering the single patient habitus to allow adequate penetration of the ultrasound beam and optimize the acoustic window [108]. Power and color Doppler are helpful in assessing vascular structures and any signs of vascularization of intraparenchymal presumed alterations [108].

Each observation should be assessed in three dimensions in the longitudinal and transverse plane, reporting their precise location and anatomical relationships. Optional cine clips may facilitate the comparison in follow-up exams [108].

#### 2.1.2. Ultrasound Category and Visualization Score

The US-LI-RADS algorithm includes both an evaluation score of the observations and an analysis of the quality of the images. The three possible categories (US-1, US-2 and US-3) should be applied to the entire examination, and each category corresponds to a subsequent clinical management [104,105,106,107,108].

The US-1 category includes the detection of clear benign formations (simple cysts, focal areas of fat sparing or hemangiomas), leading the patient to routine follow-up in 6 months [109].

The US-2 category is assigned when not definitely benign <10 mm formations are detected. Close US follow-up at 3–6 months is recommended to intercept an overthreshold growth (>1 cm), which requires further diagnostic characterization. The stability or subthreshold size in two years, redeploy the patient in the US surveillance program every six months [109].

US-3 corresponds to a positive examination for lesions >10 mm that are probably HCC. In these circumstances, a contrast-enhanced CT, MRI or US is mandatory [109].

During the examination, the radiologist should also pay attention to identifying a new thrombus in a vein, which could be a sign of tumor invasion. The second component of US-LI-RADS is the visualization score, which is an assessment of three categories (A-B-C) of the overall quality and/or perceived sensitivity of the exam [104,109]. Although the visual score should be used to indicate the expected level of sensitivity of a screening and surveillance US examination, it does not directly influence patient management and remains open territory for research [109].

### 2.2. CT/MRI LI-RADS

**Figure 3:**
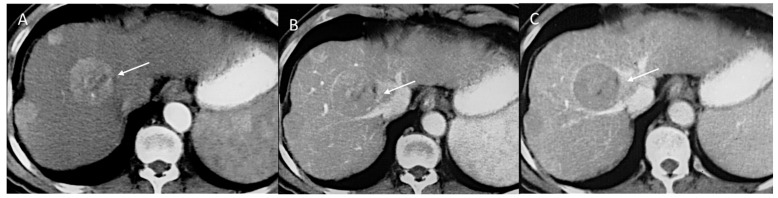
HCC on VIII seg. CT assessment. The lesion (arrow) shows APHE during arterial phase (**A**) of contrast study with washout in portal phase (**B**) and capsule appearance (**C**) in late phase.

**Figure 4:**
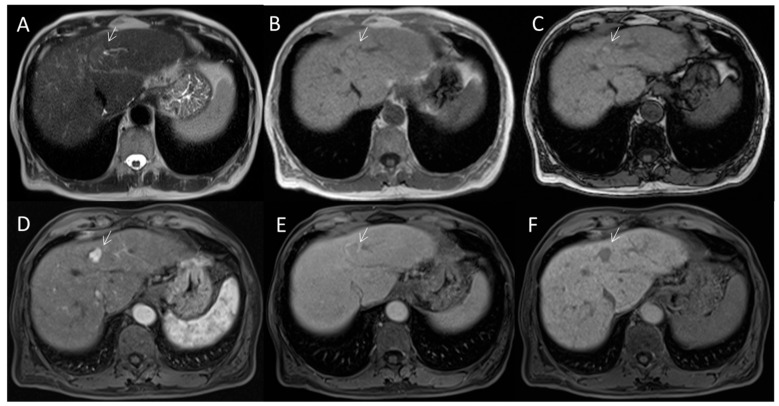
HCC on II seg. MRI assessment. The lesion (arrow) shows hyperintense signal on T2-W sequence (**A**), hypo-iso signal on T1 sequences ((**B**): in phase and (**C**): out phase) with APHE during arterial phase (**D**) of contrast study, capsule appearance during transitional phase (**E**) and hypointense signal in EOB phase (**F**).

Accurate diagnosis and staging of HCC can be achieved by CT (Figure 3) or MRI (Figure 4) in the absence of invasive methods, when precise and stringent criteria are applied [110,111,112,113,114,115,116,117,118,119,120,121,122,123,124,125,126,127,128,129,130]. Although MRI proves to be more sensitive in the characterization of liver lesions, particularly with hepatospecific contrast agents, there is no unambiguous indication from the pool of experts [131,132,133,134,135,136,137,138,139,140,141,142,143,144,145,146,147,148].

The choice to use CT rather than MRI remains at the discretion of individual institutions.

#### 2.2.1. CT/MRI Technique

The LI-RADS guidelines provide information on minimum technical requirements for CT and MRI examinations to be considered reliable. CT examinations should be performed on multidetector devices (≥8 detectors), while a magnetic field of at least 1.5 T is indispensable for MRI [67]. Intravenous contrast medium administration and a multiphasic acquisition are fundamental. Three phases of acquisition—arterial, portal and late phases—are necessary to comply with the LI-RADS requirements [67]. In particular, the arterial phase could be well timed, sufficiently late, to detect one of the hallmarks of HCC, namely the arterial-phase hyperenhancement (APHE). The late phase is acquired at 2–5 min, with some differences when a hepatospecific contrast agent is employed. When using gadoxetate disodium, no conventional late phase is obtained; instead, images are acquired in the transitional and hepatobiliary phases at 2–5 min and 15–20 min after injection, respectively [67]. If gadobenate dimeglumine is used, a conventional delayed phase is usually acquired at 2–5 min with optional imaging of the hepatobiliary phase at 1–3 h after injection [67].

In-phase and out-of-phase T1-weighted basal sequences and T2-weighted sequences (with or without fat suppression) prior to contrast injection are necessarily integrated into the MRI study, while diffusion-weighted imaging (DWI), subtraction sequences and hepatospecific phase images are optionally conducted [67].

Although there is no guidance on the assessment of the severity of artifacts, the radiologist should assign an LR-NC category (LI-RADS not categorizable), when an observation cannot be fully assessed due to missing or degraded images [67].

#### 2.2.2. CT/MRI Categories

LI-RADS includes eight diagnostic categories (-LR), reflecting the probability that an *observation* is benign, HCC, a neoplasm other than HCC or a tumor in a vein (TIV) [67].

The algorithm is built to guide the radiologist through a stepwise process, recognizing both major and ancillary features.

**Figure 5:**
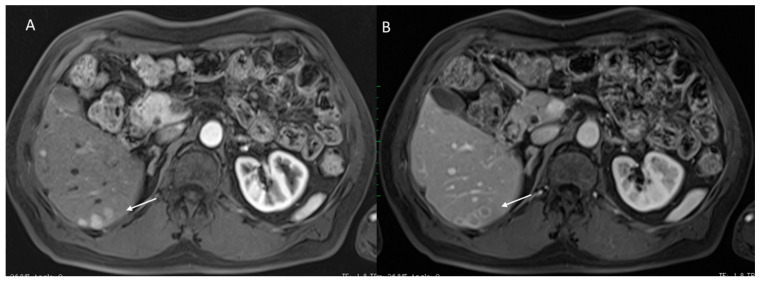
HCC on VI seg. MRI assessment. The lesion (arrow) shows APHE during arterial phase (**A**) with washout and capsule appearance (**B**) during portal phase of contrast study.

Currently, LI-RADS major imaging criteria include APHE (Figure 5), washout, capsule appearance, threshold growth and size [67].

**Figure 6:**
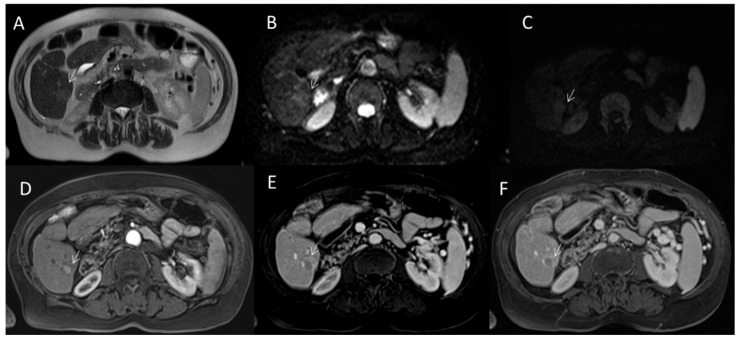
HCC on VI seg. MRI assessment. The lesion (arrow) shows hyperintense signal on T2-W sequence (**A**), restricted diffusion on DWI sequences ((**B**): b50 s/mm^2^ and (**C**): b800 s/mm^2^) with APHE during arterial phase (**D**) of contrast study, with washout and capsule appearance during portal (**E**) and late phase (**F**) of contrast study.

While the major criteria should always be satisfied, ancillary features, such as restricted diffusion (Figure 6), could be used at the discretion of the radiologist to increase or decrease the category, except to upgrade an *observation* from LR-4 to LR-5 [149,150,151].

The categories LR-1 (definitely benign) and LR-2 (probably benign) include simple cysts or nodules, <20 mm in size, that do not show features of malignancy. LR-3 (intermediate likelihood) refers to some perfusions or true nodules with one or two major malignant features. Specifically, *observations* <20 mm could be categorized as LR-3 if they have APHE as the sole major feature. *Observations* lacking APHE are categorized as LR-3 if they have a size <20 mm and ≤1 additional major feature or size ≥20 mm and no additional major features. In these circumstances, it should be indicated to repeat enhanced diagnostic imaging in 3 to 6 months [67]. The LR-4 observations are probable HCCs. The LR-4 category includes *observations* <10 mm with nonrim APHE and ≥1 additional major feature; 10 to 19 mm *observations* with nonrim APHE and “capsule” as the only major feature; *observations* ≥20 mm with nonrim APHE and no additional major features. The LR-4 category also includes *observations* without rim APHE: *observations* <20 mm and ≥2 additional major features and *observations* ≥20 mm and with ≥1 additional major feature. According to LI-RADS guidelines, LR-4 patients are referred for biopsy, presumptive treatment or follow-up, depending on the multidisciplinary team’s decision [67].

Malignancy categories include LR-5 (definitely HCC), LR-M (malignant lesion other than HCC) and LR-TIV (malignancy with tumor in vein) [67].

APHE and size ≥10 mm are required for LR-5 categorization. The LR-5 category includes *observations* >20 mm with ≥1 additional major feature and 10 to 19 mm *observations* with ≥2 additional major features. *Observations* measuring 10 to 19 mm can also be categorized as LR-5 if they have only one additional major feature: either nonperipheral “washout” or threshold growth. In the absence of diagnostic doubt, pathologically confirmed lesions and benign lesions of non-hepatocellular origin do not require LI-RADS classification.

**Figure 7:**
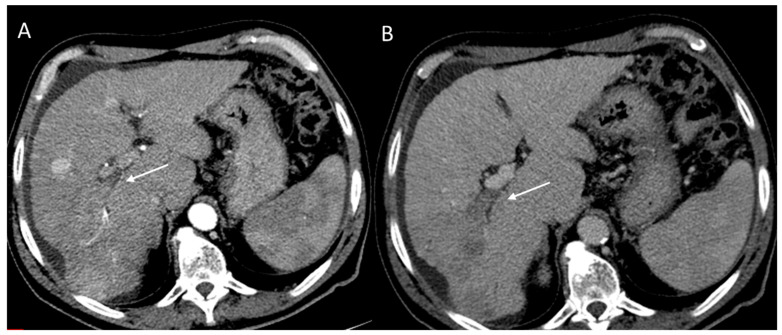
Tumor in vein. Portal thrombosis CT assessment during arterial (**A**) and portal (**B**) phase of contrast study.

CT and MRI also contribute to the staging of liver disease, particularly to the evaluation of possible macrovascular invasion, which falls into the LR-TIV category (Figure 7) and contraindicates the curative surgical approach [13,152].

In addition to the direct identification of vascular invasion by the tumor mass, findings suggestive on imaging of locally invasive HCC are the presence, within an occluded vein, of arterial neovases manifesting as thin, punctuated hyperenhancing “threads and stripes”. Another aspecific sign of TIV is the increased diameter (23 mm or more) of a thrombosed main portal branch [153]. This last finding could be carefully interpreted, as although it may suggest a tumor, it could be appreciated in a simple bland thrombus, which is frequently seen in cirrhosis. A correct differential diagnosis is critical for staging. Indeed, tumor extension to the main portal trunk or contralateral liver lobe is associated with worse overall survival in HCC patients [154].

#### 2.2.3. HCC Diagnosis

On imaging, HCC reveals a distinctive dynamic vascular pattern of APHE followed by washout in portal-venous phase. Due to its high specificity, this temporal enhancement hallmark is incorporated in all current diagnostic systems. As emerged from a recent meta-analysis, the APHE and washout are the strongest independent imaging features associated with HCC [155]. APHE is defined as the presence of nonrimlike enhancement of an *observation* that is unequivocally of higher intensity/attenuation than background liver [155]. This appearance is referable to the high arterial flow in advanced HCC due to angiogenesis and formation of nontriadal or unpaired neoarteries [156].

Among the LI-RADS major features, APHE shows the highest sensitivity (85%) for progressed HCC, but it is characterized by a low specificity (57%) [157]. Indeed, this feature may also be seen in benign entities such as hemangiomas or perfusion alterations, premalignant lesions such as dysplastic nodules or small non-HCC tumors [158]. Holland et al., in 46 patients with cirrhosis who underwent MRI before liver transplantation, noticed that most of the hypervascular lesions in the arterial phase (93%) were actually benign formations [159].

These findings were confirmed by Granata et al. in 17 hyperplastic nodules that resulted hypervascular during MRI examinations in a retrospective evaluation of 70 patients with HCC [23].

A meaningful number of *observations* with APHE could potentially be incorrectly classified as HCC; therefore, this criterion alone is considered inadequately accurate [23]. The false-positive rate could be lowered by combining APHE with the “washout” feature. [23,160].

The washout of an *observation* refers to the progressively hypointense/hypodense appearance compared to the surrounding liver parenchyma during the portal-venous phase and the delayed phase [67,161].

This contrast-enhancement behavior corresponds to a reduced portal supply, increased cellularity and reduced extracellular volume of the tumor.

APHE and washout appearance are not a feature exclusive to HCC and could be observed in cirrhotic or dysplastic nodules, raising the issue of specificity when applied as a sole criterion on liver *observations* [162,163,164,165,166,167,168,169,170,171,172].

Combining detection of APHE and washout could significantly increase specificity, reaching values close to 100% when detected in high-risk patients with nodules ≥ 20 mm [173].

This advantage is still associated with poor sensitivity, especially in smaller-sized lesions, showing inconspicuous washout [174].

The capsule appearance is the other major criterion that may improve the performance of the LI-RADS algorithm. The “capsule” presence is a distinctive sign of advanced HCC, with the highest specificity value among all major features (90–96%) [23,175]. However, this tumor appearance often coincides with the APHE/washout pattern, limiting its added value in terms of sensitivity [176].

Favoring specificity is positively accepted in Western countries, where the main purpose is to avoid false positives in order to select patients for liver transplantation. On the other hand, in Asian countries, locoregional therapies are often employed in the first instance, and therefore HCC should be intercepted at an early stage. Since LI-RADS is proposed as a universal guide in HCC management, the problem of sub-optimal sensitivity should be solved [177].

#### 2.2.4. Ancillary Features

Ancillary features (AFs) could play a decisive role. Although these imaging criteria alone preclude definitive diagnosis of HCC, they provide additional data for tumor characterization, improving the sensitivity rate of major features. The importance of AFs applied especially to MRI emerges from the literature. Between all AFs, the hypointensity in the hepatospecific phase (HPH) appears independently associated with the diagnosis of HCC [178].

HPH has shown to increase sensitivity in liver *observation* assessment, even the small ones (10–19 mm). This last clinical scenario is particularly challenging, reaching a failure rate of 87% when only major criteria are identified in small liver lesions [179].

Vernuccio et al. recorded a sensitivity of 84% applying HPH to LR-3 small *observations* with APHE upgraded to the LR-4 category [180]. This sensitivity value resulted considerably higher compared to those of LI-RADS major criteria (from 0% to 35%) [179].

The signal of an *observation* in the hepatobiliary phase depends on the expression of the OATP8 transporter, which provides the contrast agent uptake. The OATP8 expression declines during hepatocarcinogenesis, and therefore the assessment of signal intensity in the hepatobiliary phase may guide the detection and characterization of hepatocellular nodules in the cirrhotic liver. The HPH could also be seen in non-hepatocellular benign lesions such as hemangiomas or non-HCC malignancies due to the physiological absence of the OATP8 expression. Therefore, HPH is not sufficiently specific to be used as the sole criterion, but as shown in several studies, it could be associated with more AFs in order to overcome this limitation [180,181,182,183,184,185,186,187,188]. Cannella et al. observed an increase in specificity (from 86.1% to 96.2%) for HCC detection, combining HPH with three or more AFs favoring malignancy in 155 patients in LR-3 and LR-4 LI-RADS categories [189].

Additionally, Lee et al. observed that the application of AFs both of malignancy in general (mild–moderate T2 hyperintensity and HPH) and favoring HCC in particular (nonenhancing “capsule” and mosaic architecture) to the LR-4 category assigned on the basis of the major criteria significantly increases the sensitivity in the diagnosis of HCC (69.4–76.9% compared with the standard LR-5 66.2%), without impairing specificity (95.3–96.5%) [190].

Several studies have focused on the application of gadobenate dimeglumine (Gd-BOPTA) in the diagnosis of HCC and its possible advantages as a major feature in tumor assessment. Although the diagnostic performance and liver parenchymal enhancement observed during the hepatobiliary phase seem similar for both gadoxetic Gd-EOB and Gd-BOPTA [191,192], some data suggest that Gd-BOPTA exhibits a higher maximum enhancement value of the hepatic artery, portal and hepatic veins during the respective vascular phases [193].

Therefore, these peculiarities could be useful when applying the LI-RADS algorithm. Cortis et al. demonstrated increases in sensitivity from 53.2% to 75.8% for lesions of ≥20 mm and from 53.5% to 62.1% for lesions of 10–19 mm when HPH on Gd-BOPTA was included as an additional major feature to the typical APHE/washout pattern in cirrhotic patients with hypervascular HCC [194]. According to this evidence, Zhang et al. recorded an increased sensitivity in HPH on Gd-BOPTA inclusion as a major feature, without impact on specificity. Furthermore, the authors surprisingly proved that HPH on Gd-BOPTA could even replace the enhancing capsule as a major criterion in the LI-RADS algorithm, with sensitivity improvement, especially for lesions 10–19 mm in size [195]. Further studies are needed, but HBP images on Gd-BOPTA MRI could be considered an important tool for the HCC characterization of any size.

#### 2.2.5. LR-M Category

**Figure 8:**
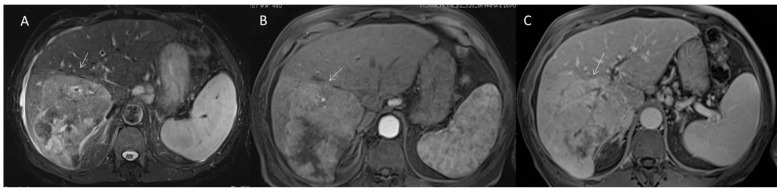
CCA on VII seg. The lesion (arrow) shows targetoid appearance in T2-W sequence (**A**) with nonrim APHE (**B**) and progressive contrast enhancement during late (**C**) phase of contrast study.

**Figure 9:**
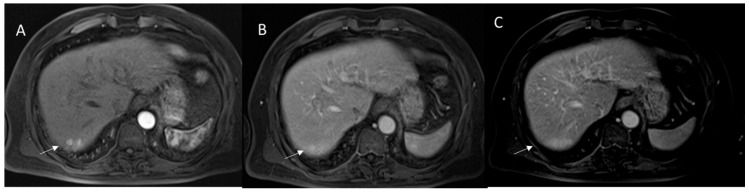
cHCC-CCA on VII seg. The lesions (arrow) show APHE during arterial phase of contrast study (**A**) without washout in portal phase (**B**) and progressive contrast enhancement during late phase (**C**) of contrast study.

Intrahepatic cholangiocarcinoma (iCCA) (Figure 8) and combined hepatocellular-cholangiocarcinoma (cHCC-CCA) (Figure 9) could develop in a cirrhotic liver displaying HCC-like characteristics [12,16].

In LI-RADS, the LR-M category collects all those *observations* with features suggestive of malignancy but missing major criteria specific for HCC (classic APHE and washout/capsule appearance). The LR-M features include: rimlike APHE, peripheral washout, liver surface retraction, adjacent biliary obstruction, mixed pattern and infiltrative margin [67].

In several studies, the rimlike APHE emerges as the most common sign suggesting malignancy other than HCC, being present in 50–84% of examined lesions. The second most frequently encountered feature resulted a delayed or progressive central enhancement during PVP or DP after extracellular agent injection, observed in 42–96% of liver *observations* [196]. Regarding the washout appearance, the literature is unclear, given the lack of distinction between peripheral lesion washout and HPH. However, these limitations are of minor importance since “washout” is described in a small percentage of non-HCC cases (4–6%) [197,198,199].

Another ancillary feature associated with the LR-M category is represented by a mild–moderate T2 hyperintensity. Cannella et al. found that a peculiar signal in the T2 sequences, the “targetoid” appearance associated with tumor vascular involvement, could be a key sign of non-HCC primary liver carcinomas (PLCs) [200]. Researchers retrospectively analyzed 165 iCCAs and 74 cHCC-CCAs, with 136 HCCs as controls. All lesions were pathologically proven and prior imaged on contrast-enhanced CT or MRI. Combining targetoid appearance on T2-weighted images and tumor vascular involvement showed high specificity (92–100%) for the diagnosis of non-HCC PLCs, with a good inter-reader agreement [200]. These results are in line with previous studies, which stressed the role of T2-weighted appearance in iCCA detection. In particular, Sheng et al. proved that dynamic enhancement patterns and T2-weighted images signal were the most important MRI discriminants between ICC and atypical small HCC (≤3 cm) in high-risk patients [201].

LR-M features defined by LI-RADS are most closely associated with the imaging appearance of ICC as the second most frequent neoplasm in cirrhotic liver [202]. Histopathologically, ICC presents a central fibrotic core of imbibed stroma and a predominantly peripheral cellularization/vascularization. This inner stratification reflects in a so-called “targetoid” enhancement pattern on imaging, with a peripheral arterial hyperenhancement in a rimlike aspect, followed by a peripheral washout with a progressive and delayed enhancement of the central core [203,204]. The same targetoid pattern could be appreciated in diffusion-weighted imaging (DWI), in which the outer layer shows more restriction than the central zone. The targetoid organization is also documented during the hepatobiliary phase on MRI: a central retention of contrast, less intense of background liver, associated with a peripheral irregular rim of hypointensity [204]. However, as demonstrated by Kim et al., the contrast enhancement patterns of ICC in cirrhotic liver depend on tumor size. Small ICCs (<3 cm) may not exhibit the typical targetoid vascular pattern, with a difficult differential diagnosis from HCC [205]. Furthermore, the targetoid appearance is not the exclusive prerogative of ICCs. Lee et al. observed that the targetoid pattern showed the highest sensitivity (75.8%) to correctly identifying cHCC-CCA as LR-M in a group of 99 patients with pathologically proven cHCC-CCA or HCC [206]. This evidence was confirmed by Choi et al. Researchers observed in 194 patients with cirrhosis and surgically proven single primary liver cancer that although the targetoid mass features on Gd-EOB were more common in ICCs (39–59%), it could be detected even in cHCC-CCs (9–21%) and HCCs (1–3%) [207]. Therefore, a mass displaying any of the features of dynamic contrast enhancement, hepatobiliary phase imaging or DWI should be generally categorized as LR-M.

In the literature, the targetoid appearance on HPH, the presence of rimlike APHE and peripheral “washout” would seem sufficiently distinctive of ICCs [208,209]. However, it should be emphasized that these are not definitive data, considering that most studies focused on the differential diagnosis between ICC and HCC have compared the characteristics of ICC with atypical HCC in a non-targeted population.

To date, only a few publications have described the imaging appearance of cHCC-CCA and the features that can be distinguished only in these lesions compared to HCC or ICC [12,16]. Several studies have evaluated the possibility of differentiating cHCC-CCA from ICC through the different contrast enhancement patterns. Park et al. divided 82 surgically confirmed cHCC-CCs lesions into subgroups according to vascular enhancement pattern on Gd-EOB-MRI [210]. Forty-eight lesions showing nonrim APHE were assigned to the hypervascular group, while thirty-four lesions demonstrating rim, peripheral or isoenhancement were assigned to the nonhypervascular group. On pathological specimens, the hypervascular group encountered a major HCC component, poor CC elements of pathological analysis and was associated with increased overall survival after curative surgery than the other group [210]. In a subsequent study, Kim et al. identified in cHCC-CCAs an intermediate-form APHE, characterized by an irregular distribution with some areas thicker than others. This type of APHE seemed able to distinguish cHCC-CCAs from ICCs [211].

Regarding other liver tumors, Granata et al. [16] assessed the diagnostic performance of LI-RADS in not-at-risk-of-HCC populations, showing that high diagnostic accuracy was obtained by LI-RADS classification between malignant and benign lesions. The presence of AFs could help the radiologist toward a correct diagnosis [16]. However, LI-RADS has a low capability to identify borderline lesions as BT-IPNB and hepatic adenomatosis as benign lesions due to oxaliplatin [16].

### 2.3. CEUS-LI-RADS

CEUS is a US examination widely accepted in many clinical contexts, performed by injecting intravenously microbubble-based contrast agents (USCAs). USCAs are small (from 1 to 10 μm) biodegradable particles of low-solubility gases (e.g., perfluoropropane, perfluorocarbon or sulfur hexafluoride) covered by a flexible lipid shell [212,213,214,215,216,217]. This type of contrast agent is confined within the intravascular space (blood pool agents) and almost completely exhaled after 20 min from the injection. Through the application of a specific US imaging mode, the microbubbles produce an echo pulse that is evidenced by tissue signal removal [218].

ACR incorporated CEUS into the LI-RADS system in 2016, driven by working groups of hepatologists and radiologists, who have demonstrated the diagnostic accuracy of CEUS in the characterization of focal liver lesions [67]. Albeit with some substantial differences from the other diagnostic algorithm, the CEUS-LI-RADS model allows a dynamic classification of hepatic focalities in different categories. CEUS provides a real-time image of the vascular behavior of intercepted lesions, eliminating the concept of observation applied in the other fields of the LI-RADS system.

Otherwise, CEUS is a focused exam, being inadequate for staging and response to therapy, lacking the panoramic and standardized visualization of CT/MRI. Considering these premises, CEUS-LI-RADS introduces different diagnostic features, requiring its own algorithm with distinctive unique properties [219].

#### 2.3.1. Technique

US equipment should comprise contrast-enhanced imaging setting, which generally includes a dual-screen and on-screen chronometer.

Prior to enhanced imaging, baseline scans are performed to assess the target nodule, choosing the optimal position for the patient in order to reduce out-of-plane motion from breathing. Using the B-mode image as a guide, the probe is positioned over the lesion on both screens simultaneously to facilitate enhancement characterization [217]. Then, a contrast agent is injected into an antecubital vein using a ≥ 20 G catheter, followed by a saline flush. Currently, there are two commercially available US contrast agents for CEUS-LI-RADS: Definity^®^ (in USA, Canada)/Luminity^®^ (outside USA, Canada) and Lumason^®^ (in USA)/Sono-Vue^®^ (outside USA).

Arterial phase begins within 20 s after injection of the contrast agent and persists until 30–45 s, depending on the cardiocirculatory situation. PVP begins 30–45 s after injection and ends at 120 s, while the late phase starts 2 min after injection, lasting until the clearance of microbubbles from liver tissue [217].

It is advisable to perform the examination continuously from contrast medium injection to the peak of arterial phase enhancement, in order to intercept APHE. Conversely, PVP and LP examinations are executed intermittently (every 30 sec) to minimize the destruction of microbubbles, allowing one to observe the late washout [217].

Finally, a survey recording is recommended from the contrast vascular input until the peak of APHE.

#### 2.3.2. CEUS-LI-RADS Categories

As in the CT/MRI model, eight diagnostic categories with related imaging work-up suggestions cover the spectrum of lesions possibly discernible at CEUS.

Nodules are classified based on major criteria: size (<10, ≥10 <20 mm, ≥20 mm), the APHE appearance and the presence and type of washout.

CEUS LR-1 and 2 categories correspond to definitely and probably benign observations, respectively. LR-1 comprises formations with unequivocally benign contrastographic behavior, whereas LR-2 is assigned to isoenhancing lesions (solid nodule <10 mm/nonmasslike formations of any size) and LR-3 nodules stable in size for ≥2 years [219,220].

Patients with CEUS LR-1 and LR-2 can resume routine 6-month US surveillance, without the need for further diagnostic investigations [219].

LR-3 (intermediate probability of malignancy) includes solid nodules ≥ 10 mm with isoenhancement in all phases, nodules <20 mm showing late washout onset (≥60 s) and a mild degree of washout. CEUS LR-3 also includes nodules <10 mm, which show only APHE as a vascular criterion. Guidelines suggest repeating the examination with contrast administration within 3–6 months, but in selected cases, the multidisciplinary team could require a biopsy [219].

The LR-4 categories (probable HCC) include nodules ≥10 mm with APHE and no appreciable washout and those with APHE, late washout onset (≥60 s) and a mild degree of washout but measuring <10 mm. Finally, also focalities ≥ 20 mm with no APHE but with late washout onset (≥60 s) and a mild degree of washout may also be characterized as CEUS LR-4 [219].

All these conditions should undergo bioptic assessment or short-term (<3 months) imaging follow-up, relying on the multidisciplinary discussion decision [219].

The LR-5 nodules fulfill all major criteria and are treated as definitely HCC without biopsy confirmation [219].

CEUS algorithm also provides an LR-M class that includes not HCC malignant lesions, which usually need biopsy confirmation. The imaging features for LR-M are rim APHE, early (<60 s) washout or marked washout resulting in a “punched-out” appearance within 2 min after contrast injection. If the image quality is poor and does not allow lesion definition, the radiologist is called upon to assign the LR-NC category [219,220].

As CT/MRI, LR-TIV refers to tumor tissue in the portal or hepatic veins.

Ancillary features favoring malignancy, without specificity for HCC, include definite interval size increase, whereas definite interval size reduction or stability ≥2 years favor benignancy. Features favoring HCC, in particular, include a mosaic appearance of the nodule and a nodule-in-nodule appearance in the arterial phase [219]. These features are applied less often in the CEUS-LI-RADS algorithm, with the same limitation of the CT/MRI model in upgrading lesions from LR-4 to LR-5 [219].

#### 2.3.3. CEUS-LI-RADS vs. CT/MRI LI-RADS

CEUS enables the possibility of real-time assessment of APHE, which might be missed or misinterpreted in other imaging studies in case of inappropriate sequences/acquisition timing. This property is particularly useful in definitive LR-2 categorization of nodules, presenting dubiously HCC (LR-3-LR4), for a suspected arterial phase appearance on the CT/MRI algorithm. Among all benign focal liver formations, the assessment of hemangiomas and arteriportal shunts could result particularly challenging [221,222]. Indeed, rapidly filling hemangiomas may present as homogeneous APHE nodules on CT/MRI, mimicking HCCs. Similarly, arterioportal shunts could be perceived as nodules or could hide malignant focalities, lowering the diagnostic accuracy of CT/MRI categorization [221].

The peculiarity of CEUS in intercepting APHE is reflected positively not only in the downgrade of LR-3 or LR-4 formations but also in the definitive diagnosis of HCC, assigning the category LR-5 to probable malignant lesions [219]. Indeed, several studies have shown that CEUS could intercept APHE in nodules categorized as LR-5, formerly misdiagnosed as LR3 or LR-4 at CT/MRI examinations.

In particular, Maruyama et al. recorded that 7/27 focal liver lesions (<20 mm) characterized as non-hypervascular (APNHE) on prior contrast-enhanced CT then showed APHE at CEUS and resulted as HCCs on histological specimens [223].

As highlighted, APHE is a necessary major feature to achieve HCC diagnosis. According to the CEUS algorithm, any nodule ≥ 10 mm with APHE is classified as LR-4 or LR-5, depending on the presence or absence of the washout criterion, respectively. This assumption is based on the “B-mode nodules visibility” as a prerequisite for CEUS evaluation. As a corollary, the LR-3 category of CEUS includes more cases of HCCs compared to the CT/MRI algorithm, since the examination is based on actual nodules, whereas LR-3 formations on CT/MRI include *observations* even <10 mm that are not necessarily well defined [224].

Albeit less frequently, mass-forming ICCs are encountered during US surveillance. On CEUS, ICC often shows rim-APHE, which is an uncommon finding in HCC. Rim-APHE was a key feature in Huang et al.’s study on 228 nodules (99 ICCs and 129 LR-M HCCs) showing sensitivity and specificity for differential diagnosis with HCCs of 70.4% and 68.8%, respectively. In addition, combing the rim-APHE with elevated CA 19-9 and normal alpha-fetoprotein (AFP) values increased sensitivity to 100% [225]. Rim-APHE should not be confused with the CT/MRI capsule appearance major criterion, which could not be appreciated on CEUS due to its fibrotic nature with large interstitial spaces and low blood volume. ICC also shows an early (<60 s) and prominent washout compared to HCC, which conversely proves a mild and late washout (≥60 s) after contrast injection [219].

Therefore, the LR-M category on CEUS-LI-RADS could be confidentially assigned in the presence of rim-APHE, early washout (<60 s), marked washout visible within the first 2 min after contrast injection or any combination of these three features [219,225].

### 2.4. LI-RADS Treatment Response Algorithm

HCC treatment was traditionally based on surgical or locoregional ablation technique [226,227,228,229,230,231,232,233,234,235,236,237]. The LI-RADS treatment response algorithm (TRA) is proposed as a comprehensive approach to standardize the disease assessment after locoregional therapies for HCC, using contrast-enhanced CT or MRI [226,227]. Compared to other criteria, such as modified *Response Evaluation Criteria in Solid Tumours* (mRECIST) [238], which evaluate the patient-level response, TRA provides the viability after treatment of the individual liver lesions [227].

Considering that the management of HCC often requires multiple therapeutic interventions, this approach could guide the multidisciplinary team caring for the patient in closely monitoring the evolution of the disease or in choosing the appropriate therapy.

**Figure 10:**
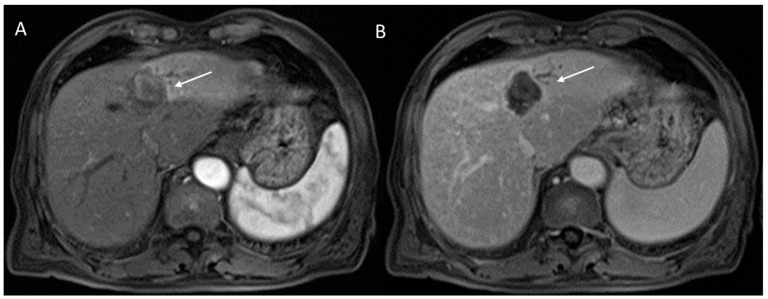
Treated HCC on II seg. Nonviable lesion. The lesion (arrow) shows necrotic appearance during arterial (**A**) and portal (**B**) phase of contrast study.

Similarly to the LI-RADS diagnostic algorithm, the TRA classifies treated HCC into three different categories: LR-TR *viable*, LR-TR *equivocal* or LR-TR *nonviable* (Figure 10), according to the post-treatment enhancement pattern [227]. LR-TR *viable* is assigned to liver observations demonstrating persistent arterial phase hyperenhancement or washout or a pattern enhancement similar to pre-treatment imaging characteristics [227]. Generally, residual neoplastic cells could be distributed in a nodular, mass-like, or in thick irregular tissue within or along the treated lesion. If the observation shows no enhancement or if only a typical post-treatment enhancement is observed, LR-TR *nonviable* is assigned. In challenging cases that are difficult to define, an intermediate category (LR-TR *equivocal*) could be assigned. The role of the intermediate category is still questioned [227].

Chaudhry et al. [239] evaluated the application of the TRA in 36 patients with 53 lesions after ablative therapy prior to liver transplantation, comparing the category assigned on imaging with the pathological finding. Assessing equivocal lesions as *viable*, the researchers observed an increase in sensitivity in predicting complete necrosis to 81–87%, compared to 40–70% when treating the same lesions as *nonviable*. Furthermore, the majority of lesions classified as LR-TR *equivocal* (five out of six (83%)) exhibited incomplete necrosis in histopathology specimens [239]. Similarly, Shropshire et al. found incomplete necrosis in 12/17 (71%) of the lesions categorized as *equivocal* after transarterial embolization [240]. Hence, the application of the LR-TR *equivocal* category would seem to increase the sensitivity in residual disease detection, intercepting some cases that might escape from a treatment program for the residual presence of HCC [240].

On the other hand, what also emerges from these studies is the unsatisfactory diagnostic accuracy of the nonviable LR-TR category in predicting a complete response to therapy. In both Chaudry’s and Shropshire’s investigations, only a low percentage of lesions assigned as nonviable were actually completely necrotic at histopathology (49% and 38–46%, respectively) [239,240,241,242].

Currently, insufficient data are available in evaluating the impact of nonviable LR-TR, in cases of minimal residual disease at pathological relief on patients’ overall survival or progression-free survival. Given the possibility of residual microfoci of disease that are difficult to distinguish on imaging after local-regional therapy, the interval for imaging follow-up should remain close, regardless of LR-TR category [67].

Although the application of TRA seems plausible in clinical practice, there are still important gaps.

For instance, an area that has not yet been sufficiently investigated concerns the evaluation of TRA performance in assessing treatment response after locoregional radiation-based therapy (transarterial radioembolization or external beam radiotherapy). Further research should be promoted to improve the algorithm, decrease the rate of LR-TR *equivocal* categorizations and provide clinical assessment of residual disease after all types of local-regional therapies.

## 3. Conclusions

LI-RADS is the most comprehensive system for the diagnosis and management of HCC. Four LI-RADS algorithms cover three individual clinical contexts: screening and surveillance, diagnosis/staging and assessment of treatment response.

CEUS-LI-RADS and CT/MRI LI-RADS algorithms could achieve confident HCC diagnosis, providing tailored management recommendations for *observations* found on US surveillance in patients considered at risk. In particular, CEUS-LI-RADS can afford a contrast-enhanced examination in absence of radiation exposure. This could be an important advantage, especially in young patients.

Currently, LI-RADS major imaging criteria should always be satisfied, while ancillary features could be used at the discretion of the radiologist to increase or decrease the category. However, the sensitivity value of these combined criteria is inadequate. Recent evidence proved that HPH on Gd-EOB-DTPA could be an important tool not only in increasing sensitivity but also in diagnosis as a major criterion. Although LI-RADS emerges as an essential instrument to support the management of liver tumors, still many improvements are needed to overcome the current limitations. In particular, features that may clearly distinguish HCC from ICC and cHCC-CC lesions and the assessment after locoregional radiation-based therapy are still fields of research. In fact, although the application of TRA seems plausible in clinical practice, there are still important gaps. An area that has not yet been sufficiently investigated concerns the evaluation of TRA performance in assessing treatment response after transarterial radioembolization or external beam radiotherapy. Further research should be promoted to improve the algorithm, decrease the rate of LR-TR *equivocal* categorizations and provide clinical assessment of residual disease after all types of local-regional therapies.

## Data Availability

Not applicable.
